# Prevalence of major digestive and respiratory helminths in dogs and cats in France: results of a multicenter study

**DOI:** 10.1186/s13071-022-05368-7

**Published:** 2022-09-06

**Authors:** Gilles Bourgoin, Marie-Pierre Callait-Cardinal, Emilie Bouhsira, Bruno Polack, Patrick Bourdeau, Clarisse Roussel Ariza, Lisa Carassou, Emmanuel Lienard, Jason Drake

**Affiliations:** 1 Laboratoire de Parasitologie Vétérinaire, VetAgro Sup, Université de Lyon, 1 Avenue Bourgelat, BP 83, 69280 Marcy l’Etoile, France; 2 Laboratoire de Biométrie et Biologie Évolutive UMR-CNRS 5558, Université Lyon 1, Université de Lyon, 69622 Villeurbanne, France; 3 Innovations thérapeutiques et résistances (InTheRes), INRAE, ENVT, Université de Toulouse, 23 chemin des Capelles, 31076 Toulouse, France; 4 BioPôle Alfort Secteur Parasitologie, Anses, INRAE, Ecole Nationale Vétérinaire d’Alfort, UMR BIPAR, Laboratoire de Santé Animale, 94700 Maisons-Alfort, France; 5 Laboratoire de Dermatologie/Parasitologie/Mycologie de LABONIRIS, Ecole Vétérinaire de Nantes site la Chantrerie d’Oniris, Université de Nantes, 44307 Nantes Cedex, CP France; 6 Elanco Animal Health, Crisco Uno, Bâtiment C, 3-5 avenue de la cristallerie, 92310 Sèvres, France; 7 Elanco Animal Health, 2500 Innovation Way, Greenfield, IN 46140 USA

**Keywords:** Internal parasites, Nematodes, Cestodes, Deworming, Risk factors, Companion animals

## Abstract

**Background:**

The local distribution of helminths in dogs and cats and the evaluation of risk of contamination represent an important challenge for veterinarians due to their effects on animal health and their potential zoonotic risk. The overall goal of this study was to estimate the prevalence of the digestive and respiratory helminths infecting client-owned dogs and cats in France.

**Methods:**

Faecal samples were collected from 414 pet dogs and 425 pet cats at 20 study sites during 2017–2018 and analysed by coproscopy. The samples included specimens collected from animals of both genders and various breeds and ages from a variety of living environments, and with different lifestyles and feeding regimes. Associations between parasitic infection and qualitative factors were explored.

**Results:**

Overall, 125 (14.9%) samples (15.2% in dogs and 14.6% in cats) were positive for at least one of the species of helminths identified. Infection rates were highest for *Toxocara canis* and *Toxocara cati* (8.5% and 11.3%, respectively), while *Toxascaris leonina* was found only in one cat (0.2%). The apparent prevalence of *Ancylostoma caninum* and *Uncinaria stenocephala* in dogs was 1.7% and 4.3%, respectively. No hookworms were found in cats. Whipworms (*Trichuris vulpis*) were identified in 2.7% of the dogs. Tapeworms (*Dipylidium caninum* and Taeniidae) were rarely found (< 1% in dogs and < 3% in cats). The prevalence of *Angiostrongylus vasorum* *Crenosoma vulpis*, and *Strongyloides stercoralis* in dogs, *Aelurostrongylus abstrusus* in cats and *Eucoleus spp.* / *Capillaria spp.* in both dogs and cats was < 1%. Significantly higher fecal parasite emission rates were identified in young individuals, in animals with outdoor access, in animals living in the countryside and in intact animals (especially in cats). In addition, cats not fed exclusively with commercial diets and living with other animals (dogs and/or cats) were at higher risk for parasites. For dogs, hunting/herding and walking off-leash were found to be additional risk factors. Furthermore, pets with no reported history of deworming or dewormed > 1 year before the study were positive for parasites significantly more often than pets dewormed < 1 year before study participation.

**Conclusions:**

The overall prevalence of helminths (some of which are zoonotic), the risk factors and the reportedly low deworming frequencies identified in this study (20.5% animals having never been dewormed and only 26.4% dewormed ≥ 3 times/year) illustrate the need for improving pet owners’ adherence to anthelmintic guidelines in France.

**Graphical Abstract:**

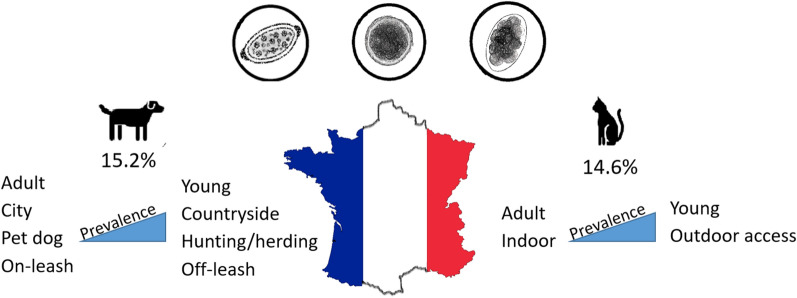

**Supplementary Information:**

The online version contains supplementary material available at 10.1186/s13071-022-05368-7.

## Background

Internal parasites of pets are of significant importance, not only because of their adverse effects on the health of dogs and cats but also due to their zoonotic potential. Helminth infections in dogs and cats can lead to a wide variety of clinical conditions, including gastrointestinal or respiratory signs, coagulopathies, neurological disorders, anaemia, dermatitis, thinning and decreased body condition and impaired performance. Severe cases can be fatal, especially in young or immunocompromised animals [1, 2]. Furthermore, *Toxocara canis* and *Toxocara cati* are considered important gastrointestinal parasites in companion animals due to their relatively high prevalence and adverse effects on animals and because they may cause visceral and ocular larva migrans as well as neurological signs or may even impart a potential booster effect on atopy in humans [1, 3, 4]. Other commonly mentioned zoonotic helminths from carnivores include, for example, *Ancylostoma caninum*, which may be responsible for human cases of cutaneous larva migrans, *Echinococcus* spp. cestodes involved in the cystic and alveolar echinococcosis, *Dipylidium caninum* and *Dirofilaria* spp. [2, 5].

Because of the pathogenicity and zoonotic risks of the canine and feline helminths, several epidemiological studies have been conducted worldwide over the past 30 years, of which about 20 were conducted in France. Half of these French surveys covered relatively small populations of dogs and/or cats (< 500 animals) and only focused on one type of parasite. Only three were prospective and multicentric, involving at least two different French regions [6–8], and only one study (published in 1997) included both dogs and cats. Consequently, updated data are desired.

The objectives of this study were to estimate the prevalence and diversity of digestive and cardio-respiratory helminths using coproscopic methods on samples collected from dogs and cats in France and to identify the main risk factors associated with their presence in companion animals.

## Methods

### Study area and study population

This multicenter survey was conducted in France at 20 different study sites, including the four National Veterinary Schools and 16 veterinary clinics distributed in 18 French departments spread across the country, between November 2017 and July 2018. Each study site enrolled client-owned pets living within the corresponding geographic area. To be eligible for inclusion, the animals should not have (i) left their department within the last 6 months, (ii) been dewormed within the past 30 days and (iii) shown signs of disorders requiring the use of a dewormer on the day of the visit.

### Faecal sample collection and parasitological procedure

At least 5 g of faeces per animal was collected by the investigators (1 designated practitioner per participating clinic or per National Veterinary School) or by the owners immediately after defecation. Samples were then sent within 48 h at ambient temperature to the laboratory of parasitology of the designated National Veterinary School for analyses or were stored for a maximum of 72 h at 4 °C before shipment. The delay between sample collection and processing did not exceed 5 days. When appropriate, tapeworm segments visible to the naked eye were collected either by the investigator or by the owners and sent together with faecal samples at room temperature.

Faecal samples were inspected macroscopically and microscopically. To help avoid potential bias associated with the use of different methods for analysing samples between the four laboratories (National Veterinary Schools), and following recommendations in veterinary parasitology for isolating eggs of nematodes and cestodes [2, 5], each laboratory utilized the same flotation method (modified from [9]) with faeces diluted in saturated NaCl solution (specific gravity: 1.2). Following this protocol, 2 g of faeces were diluted at 1:15 in saturated NaCl solution to assay the first aliquot. A McMaster slide was filled and, after a few minutes, fully observed under a microscope. A second slide was also prepared by placing a glass coverslip on a tube filled with the remaining filtered solution for 30 min and then observed under a microscope. The total number of eggs per parasite counted in the McMaster slide was multiplied by 15 to obtain the corresponding number of eggs per gram (epg). A count of 7 epg was attributed when eggs were detected only on the second slide. The remaining faeces (maximum 25 g) were used to detect cardiorespiratory larvae using the McKenna method [10]. This method was used instead of the Baermann method as it is the more sensitive of the two methods for isolating larvae [10]. After 12–24 h, the larvae were counted and identified on a slide under a microscope. Tapeworm segments, eggs and larvae were identified under a microscope using usual morphological diagnostic criteria [11, 12].

### Statistical analysis

The primary variable was defined as the presence or the absence of at least one parasite in each fecal sample. In a first step, the Chi-square (*χ*^2^) test or the alternative Fischer’s exact test for low sample size was used to detect possible associations between parasitic infection and different risk factors. We first tested for differences among the parasitology laboratories in the four National Veterinary Schools which may result from non-uniform parasite distribution in France or differences in detecting parasites. We then tested for the effect of animal’s age (3 modalities: ≤ 6 months, 6–24 months, > 24 months of age), sex (2 modalities: male, female), number of other pets in the house (two variables: number of conspecific pets in the house, total number of dogs and cats in the house; each variable had 3 modalities: no other pet, 1–2 other pets, ≥ 3 other pets), food type (3 modalities: kibbles/wet only, kibbles/wet with extra, homemade including raw meat) and the time since last deworming (4 modalities: 1–2 months ago, 3–6 months ago, 6–12 months ago and the modality “never”, which was attributed to animals not dewormed within 365 days of study participation and for kittens/puppies that had never been dewormed) on the prevalence of parasites. We also tested for an influence of the reproductive status (2 modalities: intact, neutered/spayed), but only for individuals aged > 6 months to avoid confounding effects of age and reproductive status and because we expected that sex would influence parasite abundance mostly in sexually mature individuals. Additional variables were also considered. For cats, we tested for the influence of both the lifestyle and the living environment using a three-modality variable (indoor, outdoor access in the city, outdoor access in the countryside). For dogs, we tested for an influence of the living environment (city/countryside), the lifestyle (3 classes: indoors, outdoors with limited or unlimited contacts with other pets), activity (pet, hunting/herding), time spent outside without a leash (4 modalities: 0%, < 50%, 50% ≤ *x* < 100%, 100%) and observed coprophagy (yes/no).

In a second step, using multivariate logistic analyses, all those risk factors found to have a significant influence (*P* < 0.05) based on the Chi-square test (or Fisher test) were considered simultaneously to improve our understanding of the relationships among them. When an influence of the reproductive status was detected with the Chi-square tests, we tested for the influence of the reproductive status using the previously selected multivariate model on the data subset including individual animals aged > 6 months. Model selections for multivariate logistic analyses were performed by comparing all of the models, including the different combination of variables, using the Akaike’s information criterion corrected for small sample size (AICc). We retained the model with the lowest AICc value, and when ≥ 2 competing models had a ΔAICc < 2, we retained the simplest model according to the parsimony rule [13]. All analyses and model plots were performed using R 4.0 [14].

## Results

### Dogs

A total of 414 faecal samples were analysed from client-owned dogs. The median age of dogs included in the study was 2.4 years (95% confidence interval (CI): 0.2–14.2, range: 42 days to 16.3 years), with 79.5% of the dogs older than 6 months (Table 1). The majority of the dogs included in the study were pet dogs (92.5%, *n* = 383/414) and pure breed dogs (74.0%, *n* = 305/412). Samples were evenly distributed between genders, with 51.4% (*n* = 213/414) of samples obtained from male dogs and 35.9% (*n* = 60/167) and 53.1% (*n* = 86/162) of samples obtained from male and female dogs aged > 6 months of age being spayed/neutered, respectively. More than half of the dogs (53.1%, *n* = 211/397) lived in a closed environment where the contact with other dogs or cats was limited (indoor or outdoor limited). The majority of dogs lived in the countryside (63.2%, *n* = 261/413), and 40.4% (*n* = 167/413) of the dogs spent at least half of their outdoor time off-leash. Dogs frequently lived with at least one additional animal (dog or cat) in the same household (57.9%, *n* = 239/414). Coprophagy was reported by 19.9% of dog owners (*n* = 82/412). Most of the dogs (67.15%, *n* = 278/414) were fed exclusively with kibbles and/or wet food, and only 3.9% (*n* = 16/414) were fed with homemade food. Of the 414 dog owners, 12.1% (*n* = 50/414) reported never deworming their dog, including 31.8% (*n* = 27/85) of dogs aged ≤ 6 months, 8.9% (*n* = 9/110) aged 6-24 months and 6.4% (*n* = 14/219) aged >24 months.

**Table 1:** Sample sizes and apparent prevalence of each variable and modalities for dogs and cats, with the results of univariate statistics on apparent prevalence of all helminth’s species

Variables/Modalities	Dogs				Cats			
	* N*	Apparent prevalence (%)	[95% CI]	*P*-value	* N*	Apparent prevalence (%)	[95% CI]	*P*-value
*Age*								
≤ 6 months	85	29.4	[20.1–40.3]	***	58	34.5	[22.5–48.1]	***
6–24 months	110	16.4	[10.0–24.6]		163	18.4	[12.8–25.2]	
> 24 months	219	9.1	[5.7–13.8]		204	5.9	[3.1–10.0]	
*Sex*								
Male	213	12.7	[8.5–17.9]	NS	196	13.8	[9.3–19.4]	NS
Female	201	17.9	[12.9–23.9]		229	15.3	[10.9–20.6]	
*Reproductive status if ≥ 6 months*								
Intact	183	14.8	[10.0–20.7]	**·**	104	22.1	14.6–31.3]	***
Neutered/spayed	146	7.5	[3.8–13.1]		263	7.2	[4.4–11.1]	
*Other pets in the house*								
Number of conspecific								
- 0	262	13.0	[9.2–17.7]	NS	228	11.8	[7.9–16.8]	NS
- 1 or 2	128	18.0	[11.7–25.7]		147	17.0	[11.3–24.1]	
- 3 or more	24	25.0	[9.8–46.7]		47	19.1	[9.1–33.3]	
- NA					3			
Number of other dogs and cats								
- No	174	12.6	[8.1–18.5]	**·**	174	13.2	[8.6–19.2]	*
- 1 or 2	169	13.6	[8.8–19.7]		178	11.8	[7.5–17.5]	
- 3 or more	70	24.3	[14.8–36.0]		70	24.3	[14.8–36.0]	
-NA	1				3			
*Food type*								
Kibbles/wet only	278	15.5	[11.4–20.3]	NS	347	12.4	[9.1–16.3]	*
Kibbles/wet + extra	120	15.0	[9.1–22.7]		61	21.3	[11.9–33.7]	
Homemade incuding raw meat	16	12.5	[1.6–38.3]		16	31.3	[11.0–58.7]	
NA					1			
*Lifestyle/living environment*								
Indoor					202	6.9	[3.8–11.4]	***
Outdoor in city					63	15.9	[7.9–27.3]	
Outdoor in countryside					160	23.8	[17.4–31.1]	
*Living environment*								
City	152	6.6	[3.2–11.8]	***				
Countryside	261	20.3	[15.6–25.7]					
NA	1							
*Lifestyle*								
Indoor	17	5.9	[0.1–28.7]	NS				
Outdoor limited	211	15.6	[11.0–21.3]					
Outdoor unlimited	186	15.6	[10.7–21.6]					
*Dog's activity*								
Pet	383	13.6	[10.3–17.4]	**				
Hunting/herding	31	35.5	[19.2–54.6]					
*Time walking off-leash for dogs*								
0%	72	6.9	[2.3–15.5]	*				
< 50%	174	16.7	[11.5–23.1]					
50–100%	131	14.5	[9.0–21.7]					
100%	36	27.8	[14.2–45.2]					
NA	1							
*Coprophagy*								
Yes	82	15.9	[8.7–25.6]	NS				
No	330	14.8	[11.2–19.2]					
NA	2							
*Time since last deworming*								
1–3 months ago	148	13.5	[8.4–20.1]	*	100	10.0	[4.9–17.6]	**
3–6 months ago	116	14.6	[8.8–22.4]		93	12.9	[6.8–21.4]	
6–12 months ago	65	10.8	[4.4–20.1]		60	5.0	[1.0–13.9]	
>12 months / Never	69	27.5	[17.5–39.6]		147	22.4	[16.0–30.1]	
NA	16				25			

CI Confidence interval, *NA* data not available, *N* Sample size

*P*-values: *NS*: not significant; ·: close to significant (P < 0.1);

*, **, ***Significant difference at *P* < 0.05, *P* < 0.01 and *P* < 0.001, respectively

From the 414 samples tested in dogs, 63 (15.2%) were positive for at least one helminth, including 62 (15.0%) with nematodes and only two (0.5%) with the cestode *D. caninum* (Table 2). Among these, 12 dogs (2.9%) had a mixed infection with two or three different helminth species. The most frequent helminths were *T. cani*s (8.5%; median intensity: 84 epg, maximum: 12,400 epg) and *Uncinaria stenocephala* (4.3%; median intensity: 74 epg, maximum: 1850 epg; Additional file 1: Table S1). *Trichuris vulpis* and *A. caninum* were present in 2.7% (median intensity: 15 epg, maximum: 800 epg) and 1.7% (median intensity: 100 epg, maximum: 500 epg) of faecal samples, respectively. Other nematodes (Angiostrongylus vasorum, Crenosoma vulpis, Strongyloïdes stercoralis and Eucoleus spp./Capillaria spp.) and Dipylidium caninum were each identified in 0.2–0.5 % of the samples (Table 2).

**Table 2:** Samples and apparent prevalence for each helminth’s species in dogs and cats

Helminth infection		Dogs			Cats		
		*N*	App. Prev.	[95% CI]	*N*	App. Prev.	[95% CI]
N total samples		414			425		
Gastro-intestinal and cardio-respiratory nematodes		**62**	**15.0%**	**[11.7–18.8]**	**53**	**12.5%**	**[9.5–16.0]**
Eggs	*Toxocara canis*	35	8.5%	[5.9–11.6]	–	–	–
	*Toxocara cati*	–	–	–	48	11.3%	[8.4–14.7]
	*Toxascaris leonina*	0	0.0%	[0.0–0.9]	1	0.2%	[0.0-1.3]
	*Ancylostoma caninum*	7	1.7%	[0.7–3.5]	–	–	–
	*Ancylostoma tubaeforme*	–	–	–	0	0.0%	[0.0-0.9]
	*Uncinaria stenocephala*	18	4.3%	[2.6–6.8]	0	0.0%	[0.0-0.9]
	*Trichuris vulpis*	11	2.7%	[1.3–4.7]	–	–	–
	*Strongyloïdes *	1	0.2%	[0.0–1.3]	0	0.0%	[0.0-0.9]
	*Eucoleus *spp./*Capillaria *spp.	2	0.5%	[0.1–1.7]	4	0.9%	[0.3-2.4]
Larvae	*Angiostrongylus vasorum*	2	0.5%	[0.1–1.7]	–	–	–
	*Aelurostrongylus abstrusus*	–	–	–	3	0.7%	[0.1-2.1]
	*Crenosoma vulpis*	1	0.2%	[0.0–1.3]	–	–	–
Gastro-intestinal cestodes (eggs and segments)		**2**	**0.5%**	**[0.1–1.7]**	**12**	**2.8%**	**[1.5–4.9]**
* Dipylidium caninum*		2	0.5%	[0.1–1.7]	8	1.9%	[0.8-3.7]
Taeniidae		0	0.0%	[0.0–0.9]	5	1.2%	[0.4-2.7]
Co-infestations							
Two or more parasites detected		12	2.9%	[1.5–5.0]	5	1.2%	[0.4–2.7]
Both nematodes and cestodes		1	0.2%	[0.0–1.3]	4	0.9%	[0.3–2.4]
All helminth's parasites		**63**	**15.2%**	**[11.9–19.0]**	**62**	**14.6%**	**[11.4–18.3]**

**Fig. 1:**
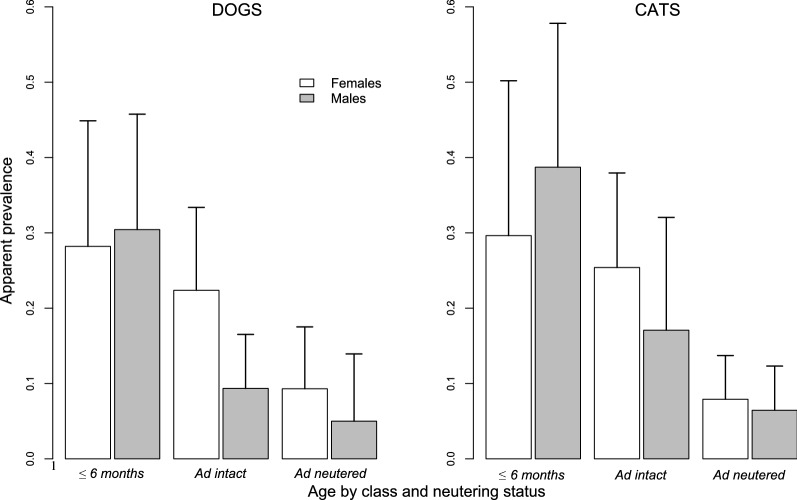
Apparent prevalence and 95% confidence interval by age and reproductive status for dogs and cats

Univariate tests demonstrated significant correlations between the apparent prevalence of parasites in dogs and age of dog (Fig. 1), living environment, proportion of time spent outside off-leash, activity of the dog (hunting/herding vs pet dog) and the time since last deworming (Table 1). Considering only dogs aged > 6 months (*n* = 329), the prevalence of parasites tended to be higher in intact compared to spayed/neutered dogs (14.8% vs 7.5%; Chi-square test,* χ*^2^ = 3.47, *df* = 1, *P* = 0.063; Table 1; Fig. 1).

All significant variables in the univariate tests were tested in multivariate logistic analysis. Based on data from 397 dogs, the selected model included the additive effects of age, activity of dogs and living environment (Table 3). The prevalence of parasites decreased with increasing age of dogs (Fig. 1), with dogs aged ≤ 6 months having a higher prevalence of parasites (odds ratio [OR]: 2.33, 95% CI: 1.15–4.87) and those aged > 24 months having a lower prevalence (OR: 0.55, 95% CI: 0.27–1.12) than dogs aged 6–24 months (Tables 1, 4). Apparent parasite prevalence was higher in hunting/herding dogs compared to pet dogs (OR: 3.1, 95% CI: 1.30–7.17) and in dogs living in the countryside compared to dogs living in city (OR: 3.17, 95% CI: 1.58–6.97).

**Table 3:** Model selection of mixed-effects models based on Akaike’s information criterion corrected for small sample size for testing the effects of different risk factors on parasitic infection in dogs and cats

Response variable	Model	*df*	AICc	ΔAICc	Weight^a^
Dogs	Age + activity + LE + off-leash	8	318.7	0.00	0.299
	Age + TSLD + activity + LE + off-leash	11	319.7	1.01	0.180
	Age + activity + LE	5	320.2	1.48	0.142
	Age + TSLD + activity + LE	8	320.5	1.75	0.125
	Age + LE + off-leash	7	320.7	2.02	0.109
	Age + TSLD + LE + off-leash	10	320.9	2.22	0.098
	Age + TSLD + LE	7	320.7	2.02	0.109
	Age + LE	4	324.5	5.78	0.017
	Age + activity + off-leash	7	328.1	9.37	0.003
	Age + TSLD + activity + off-leash	10	328.1	9.42	0.003
Cats	Age + LLE	5	281.5	0.00	0.323
	Age + LLE + food	7	282.0	0.49	0.253
	Age + LLE + TSLD	8	282.7	1.14	0.183
	Age + LLE + TSLD + food	10	284.3	2.73	0.083
	Age + LLE + NODC	7	284.7	3.13	0.067
	Age + LLE + food + NODC	9	285.6	4.08	0.042
	Age + LLE + TSLD + NODC	10	286.0	4.43	0.035
	Age + LLE + TSLD + NODC + food	12	288.0	6.43	0.013
	Age + food	5	298.9	17.40	0.000
	Age + TSLD + food	8	299.2	17.69	0.000

Only the 10 first ranked models are presented. The final selected model is shown in with underlining

*AICc (Akaike Information Criterion *Corrected) for small sample size, *LE* living environment, *LLE* lifestyle/living environment, *NODC* number of other dogs and cats, *TSLD* Time since last deworming

^a^Weight Akaike weights

**Table 4:** Model estimates of the selected models testing the influence of risk factors on parasitic infection in dogs and cats

Response variable	Variable	β ± standard error	*t*-value	*P*
Dogs	Intercept	− 2.61 ± 0.41	− 6.380	***
	Age (≤ 6 months)	0.85 ± 0.37	2.31	*
	Age (> 24 months of age)	− 0.60 ± 0.36	− 1.66	
	Activity (hunting/herding)	1.13 ± 0.43	2.61	**
	LE (countryside)	1.15 ± 0.38	3.07	**
Cats	Intercept	− 2.45 ± 0.34	− 7.29	***
	Age (≤ 6 months)	0.90 ± 0.37	2.41	*
	Age (> 24 months of age)	− 1.55 ± 0.40	− 3.90	***
	LLE (outdoor access in the city)	1.36 ± 0.48	2.84	**
	LLE (outdoor access in the countryside)	1.67 ± 0.37	4.54	***

Reference levels for each categorical variable: 6–24 months for Age; pet for Activity; 0% for the Time spent outside without a leash (off-leash);, for dogs, City for Living Environment (LE) and, for cats, indoor for lifestyle/living environment (LLE)

*, **, ***Significant difference at *P* < 0.05, *P* < 0.01, *P* < 0.001, respectively

### Cats

A total of 425 faecal samples were analysed from client-owned cats. The median age of cats included in the study was 1.8 years (95% CI: 0.2–14.9, range: 30 days to 19.0 years), with 86.3% (*n* = 367/425) of cats aged > 6 months (Table 1). The majority of cats included in the study were a European breed (87.8%, *n* = 373/425). More than 46% of the enrolled cats were males (*n* = 196/425), with 75.15% (*n* = 124/165) and 68.8% (*n* = 139/202) of male and female cats older than 6 months being spayed/neutered, respectively. More than 52% (*n* = 223/425) of the cats had free access to the outside. The majority of the cats (57.2%, *n* = 243/425) lived in the countryside. Most of the cats (58.8%, *n* = 248/422) lived with other companion animals (cat or dog). The majority of the cats (81.8%, *n* = 347/424) were fed exclusively with kibbles and/or wet food. Of the cats included in the study, 28.8% (*n* = 122/423) had never been dewormed before inclusion in the survey, of which 70.7% (*n* = 41/58), 26.4% (*n* = 43/163) and 18.8% (*n* = 38/202) were aged ≤ 6 months, 6–24 months and > 24 months, respectively. Most of the cats aged > 6 months were treated 1–2 times/year (39.0% [*n* = 62/159] and 60.7% [*n* = 122/201] for cats aged 6–24 months and > 24 months, respectively).

Parasites were detected in 62 (14.6%) feline faecal samples, including 53 (12.5%) with nematodes and 13 (3.1%) with cestodes (Table 2). Five cats (1.2%) had a mixed infection with two or three different helminth species, with four of these cats (0.9%) infected by both nematodes and cestodes. *Toxocara cati* was the most common helminth found in cat fecal samples (11.3%; median intensity: 500 epg, maximum: 6000 epg; Additional file 1: Table S1) whereas *D. caninum*, Taeniidae, *Eucoleus* spp./Capillaria spp.,* Aelurostrongylus abstrusus* and *T*. *leonina* were detected in 0.2–1.9% of the faecal samples.

Using univariate tests, we found significant correlations between the prevalence of parasites and age of cat (Fig. 1), presence of other animals (cat and/or dog) at home, living environment/lifestyle, food type and time since last deworming (Table 1). Considering only cats aged > 6 months (*n* = 367), we observed a significantly lower prevalence of parasites in spayed/neutered cats compared to intact cats (Chi-square test,* χ*^2^ = 14.87, *df* = 1, *P* < 0.001; Table 1; Fig. 1).

We therefore included all of these significant variables in a multivariate logistic model. Based on data from 397 cats, the selected model (AICc weight: 0.323) included the age of cats and their living environment/lifestyle to explain parasite prevalence (Table 3). We observed a significant decrease of parasite prevalence with increasing age of cats, with cats aged ≤ 6 months having a higher prevalence (OR: 2.46, 95% CI: 1.18–5.15) and cats aged > 24 months having a lower prevalence (OR: 0.21, 95% CI: 0.09–0.45) compared to cats aged 6–24 months. Cats with access to the outdoors in both city and countryside areas had a higher prevalence of parasites compared to cats without access to the outdoors (OR: 3.89, 95% CI: 1.50–9.96 and OR: 5.30, 95% CI: 2.64–11.26, respectively), and no differences were observed between cats with access to the outdoors in either city or countryside areas (Wald test, *Z* = 0.72, *P* = 0.468).

Using a data subset that included the previously selected variables in the multivariate model (age and living environment/lifestyle) and only cats aged > 6 months (*n* = 367), we tested the influence of the neutered/spayed status on parasite prevalence. The best model included the additive effect of the reproductive status (likelihood ratio test,* χ*^2^ = 7.64, *df* = 1, *P* = 0.006) with a significantly lower parasite prevalence in neutered/spayed cats compared to intact cats (OR: 0.33, 95% CI: 0.14–0.73, *P* = 0.007).

## Discussion

The aim of the present study was to estimate the current prevalence of major digestive and respiratory helminths in client-owned dogs and cats in France and to explore associations between parasitic infestation and qualitative factors. The results indicate that *Toxocara cati* in cats and *Toxocara canis* and *U. stenocephala* in dogs were the most common helminths detected in the faecal samples, whereas whipworms (*Trichuris vulpis*), S*trongyloïde*s *stercoralis*, E*ucoleu*s spp./*Capillaria* spp., the French heartworm *Angiostrongylus vasorum*, the lungworms *Aelurostrongylus abstrusus* and *Crenosoma vulpis*, and tapeworms (*D. caninum* and Taeniidae) were less commonly found. Significant correlations were observed between infection and the following criteria: age and deworming habits for dogs and cats; reproductive status, food type, presence of other animals in the house and living environment/lifestyle for cats; and living environment, dog’s activity and time spent outside off-leash for dogs.

The detection of endoparasites was based on standardized coprological analyses performed by trained people in expert centers. The techniques and protocols used in each laboratory were in accordance with standard guidelines and routinely conducted for parasitological diagnosis. However, coproscopical analyses have a low sensitivity for some parasites and, consequently, some limitations of the methodology and analysis may have resulted in underestimation of the reported prevalence [15]; these include:

(i) Some parasites may have not been detected because they were still immature (sample collected during the pre-patent period, before the development of mature adults) or because the shedding of eggs or tapeworm segments in the faeces was intermittent for some parasites. In this study, faecal samples were collected at a single time-point for each animal in order to avoid onerous practical constraints for the owners and to limit the risk of reduced participation. To limit biases associated with intermittent shedding of propagules and improve detection probability, faecal sampling could have been performed on 3 consecutive days [6, 16].
(ii) Faecal samples collected in the private clinics were shipped at ambient temperature within 48 h, and analysed within 5 days after collection (stored at 4 °C). During shipment, some worm eggs may have hatched and, consequently, may be not detected in the fecal float, resulting in a false negative result.
(iii) Many different procedures and techniques are used, each with their own advantages and limitations. The methods most frequently used to recover parasite eggs are flotation techniques that rely on the differences in the specific gravity of the egg(s), fecal debris and flotation solution. The specific gravity of most parasite eggs is between 1.05 and 1.23 [17]. The saturated sodium chloride (NaCl; specific gravity: 1.20) used in this study is effective for recovering *Toxocara canis*,* A. caninum *and *Trichuris vulpis* eggs*.* For all three of these parasites, while an additional centrifugation step should have isolated a significantly higher fecal counts compared with the simple fecal flotation method, the specific gravity of the selected flotation solution and the absence of centrifugation do not seem to affect the number of infections detected [18, 19]. Incorporating other diagnostics strategies, including detection of fecal antigen for nematodes, would certainly have helped to improve diagnostic sensitivity in our study [20].

The diagnosis of larvae from cardio-pulmonary nematodes (e.g. *Angiostrongylus vasorum* and *Aelurostrongylus abstrusus*) can be based on several techniques, including Baermann/McKenna coproscopy, serological test detecting circulating antigens, enzyme-linked immunosorbent assays (ELISAs) for antibody detection and quantitative PCR test on bronchoalveolar lavage material. Studies comparing serological tests and the coproscopical technique of Baermann have shown that coproscopy remains a very useful tool for the diagnosis of both *A. vasorum* and *A. abstrusus* [21, 22]. In this study, the McKenna technique was used because it is easier to use and more sensitive than the Baermann method [10].

Previous epidemiological surveys performed in France on the prevalence of internal parasites in pets showed contrasting results. Comparing these studies is difficult due to their varying designs, methods used for detecting parasites, animal populations, geographical location, environment, age distribution and seasons of sample collection [7, 8, 23, 24].

In the present study, 15.2% of the dogs and 14.6% of the cats were infected by at least one of the targeted parasites, which is below the prevalence rates reported in the last national survey report from 1997 (21.6% in dogs and 17.3% in cats) [8]. In both studies, the prevalence of infection in animals aged < 1 year was relatively similar for dogs but lower for cats in the present study compared to results obtained in 1997 (27.7% vs 24.7% in dogs and 31.7% vs 23.5% in cats, in 1997 and in the present study, respectively). Ascarids (*Toxocara canis* and *T. cati*) were the most frequently found helminths (8.5% in dogs and 11.3% in cats), with prevalence rates similar to those reported in previous studies (5.4–23% for *T. canis* and 2.9–14.2% for *T. cati* [8, 23, 25]). *Toxascaris leonina* was not detected in dogs and only detected at a very low prevalence in cats (0.2%), similar to previous French reports, with the usual findings of < 1% of positive animals [6, 7, 26]. In the present study, hookworms were identified in dogs (*Ancylostoma caninum* and *U. stenocephala*, 1.7% and 4.3%, respectively), but not in cats (*Ancylostoma tubaeforme*). These results were within the range of prevalence rates previously reported for these parasite species (0.5–3.4% for *A. caninum* and 2.1–17.2% for *U. stenocephala*) in other studies conducted in France [23, 27]. *Trichuris vulpis* was detected in 2.7% of the dogs in our study, whereas prevalence rates in another survey in France reached 19% [26]. This difference may be due to only client-owned dogs being enrolled in the present study, while previous surveys included animals living in groups. The sensitivity of coproscopical methods between surveys may also have resulted in significantly different findings. Prevalence rates for tapeworms based on coproscopy generally do not exceed 3% in France [6, 7]. We detected tapeworms (*D. caninum* and Taeniidae) more frequently in cats (1.9% and 1.2%, respectively) than in dogs (*D. caninum* only [0.5%]). However, these prevalences of tapeworms might be commonly underestimated, especially due to the intermittent rectal excretion of gravid segments.

In the present study, cardio-respiratory nematodes were rarely detected in the faeces of dogs and cats (0.5% for the French heartworm *Angiostrongylus vasorum,* 0.2% for *Crenosoma vulpis* and 0.7% for *Aelurostrongylus abstrusus*). This prevalence of *A. vasorum* is lower than that reported in previous studies in France (1.1–1.3%) [25, 28] and in countries bordering France (e.g. 0.5–3.1%) [29–31] in healthy client-owned dogs, with differences accounted for according to the detection method used (antigen detection, antibody detection and/or coproscopical analyses). In France, the cat lungworm, *A. abstrusus*, is considered to be sporadic. However, in recent years, the distribution of this parasite seems to be spreading in several countries, with prevalence rates up to 20% in enzootic areas [6, 32, 33]. In our study, the other metastrongyloid, *Troglostrongylus* spp., which is responsible for severe respiratory disease in cats, was not detected whereas it has been recently reported in southern Europe [34–36].

Younger age is associated with a higher risk of internal parasitism, as observed in our study and in previous studies [37, 38]. Certain modes of transmission (e.g. trans-placental and/or trans-mammary contamination) that are exclusive to neonates and the limited immunity to parasites in young pets explain the higher prevalence of *T. canis* or *T. cati* in young individuals [6, 8].

Living environment and lifestyle are also major factors influencing parasite risk for both dogs and cats. A positive correlation between parasite prevalence and rural areas has been described for *T. cati*, *Ancylostoma* spp. and lungworms in cats [38] and *T. canis* in dogs [39]. The higher parasite prevalence in cats with outdoor access was previously reported for *T. cati* and *A. abstrusus* [6, 38, 39].

We observed that outdoor access, rural areas, hunting/herding and time off-leash for dogs are the main factors increasing the risk of parasite risk. All of these living conditions are associated with outdoor access of animals with no or limited control of animal activities from the owner when outdoors, and outdoor areas are typically larger and vegetated. In addition, wildlife is more abundant and diverse in rural and natural areas. All of these factors are expected to increase the probability of encountering infective parasite stages by dogs and cats, either on the ground or in intermediate/paratenic hosts (e.g. snails, birds, rodents).

Cats living with several other pets were significantly more infected than cats living alone or with few animals, and a similar trend was observed for dogs in our study. In a previous study, cats living with one or two other cats were not significantly more infected than cats living alone, but for higher densities of cat populations (> 3 other cats in the household), the risk for *Toxocara* infestation was significantly higher [6]. This higher observed prevalence can be due to a higher risk of contamination due to the higher number of animals in a limited environment, to a higher probability to hunt and eat prey and, also, perhaps to a lower interest or financial support for veterinary care by owners of several animals.

The food type was associated with parasite prevalence in cats in the univariate analyses, with a higher prevalence in cats not fed exclusively with commercial diets. The lowest prevalence was observed in cats exclusively fed commercial diets, and the prevalence was found to increase with the partial or full replacement of commercial diets with alternative food. Parasite prevalence exceeded 30% in cats exclusively fed homemade food and raw meat. While such a factor was not relevant in a previous study [40], it might partly be explained by the presence of infective parasite stages in raw or undercooked meat.

Intact cats more frequently harboured parasites in their faeces than spayed/neutered cats in our study. Such findings may result from lower roaming activities in neutered/spayed cats compared to intact cats, decreasing the potential risk of exposure to parasites [41], even if some authors of previous studies did not observe any difference in activity level according to reproductive status [42, 43]. In addition, intact cats are probably less medicalized than neutered/spayed cats and, therefore, deworming probably occurs less frequently (never dewormed cats aged > 6 months: 35.9% (*n* = 37/103) in intact cats and 16.8% (*n* = 44/262) in neutered cats, respectively; *P* < 0.001).

Guidelines for the control and treatment of parasites in pet animals have been proposed by the European Scientific Councel Companion Animal Parasites (ESCCAP) [44]. These guidelines include the recommendation that puppies should be treated with appropriate anthelminthics against roundworms from the age of 2 weeks, then every 14 days up to 2 weeks after weaning because of milk transmission, and then monthly up to 6 months of age. The schedule should be similar in cats, except that because prenatal infection does not occur in kittens and, therefore treatment every 2 weeks can begin at 3 weeks of age [44]. The guidelines also describe the various risk factors to help veterinarians propose a customized deworming program to pet owners.

Although the majority of pet owners give their pets anthelminthic drugs, our results show that most owners do not follow the ESCCAP recommendations [44]. Of the animals included in the present study, 32% of dogs and 23.8% of cats had not been dewormed within the 12 previous months. The proportion of animals never dewormed was the highest in animals aged < 6 months (≤ 6 months vs. > 6 months of age: 70.7% [*n* = 41/58] vs. 22.2% [*n* = 81/365] for cats; 31.8% [*n* = 27/85] vs. 7.5% [*n* = 23/306] for dogs). However, young animals were often only a few months old when recruited into the study, when they were presented to the clinic for vaccines, and their owner(s) had not received any recommendation from a vet before study recruitment. This can explain the low frequency of previously dewormed animals in the young animal group. The generally advocated four-times-a-year deworming advice was poorly implemented as only 38.9% (*n* = 37/95) and 24.1% (*n* = 81/208) of dogs and cats, respectively, aged > 2 years with outdoor access received ≥ 3 deworming treatments per year. Moreover, as suggested by the results of recent studies [45, 46], a significant percentage of dogs or cats could “benefit” from more frequent treatment or faecal analyses, as suggested by ESCCAP [44].

The results obtained in this study show that faecal samples from animals never dewormed /dewormed > 1 year ago were positive for helminths significantly more often than samples from animals dewormed within 365 days of study participation. Surprisingly, parasite prevalence was the lowest in animals dewormed 6–12 months previously. This unexpected observation along with the absence of significant influence of the time since last deworming in multivariate analyses suggest statistical biases. We cannot exclude confounding effects with age and parasitic risk, such as: (i) young pets were often never dewormed prior to the first visit to the veterinarian and enrolment in the study, but were then dewormed monthly prior to the subsequent examination; (ii) young pets have a higher prevalence of parasites and are more frequently dewormed than adult animals; and (iii) deworming frequency in pets aged > 6 months is prescribed according to parasitic risk, leading to low frequency of deworming in pets at low risk.

## Conclusion

The results of the present study confirm that roundworms (*Toxocara canis* and *Toxocara cati*) are the most common helminths found in owned pets in France and that infections with other gastrointestinal nematodes such as hookworms (*U. stenocephala, Ancylostoma caninum* and *A. tubaeforme*), whipworm (*T. vulpis*) and cestodes continue to occur regularly.

The age, reproductive status, dog’s activity, living environment, lifestyle and husbandry were risk factors associated with the helminth infection in this animal population. Furthermore, considering both the zoonotic potential of some of these parasites and the low deworming frequencies reported, veterinarians should increase the awareness of pet owners to parasite infection and encourage adherence to deworming guidelines based on the individual risk assessment and regular coproscopic examination. Parasite control programmes could notably benefit from the implementation of a “one health approach”, improving the communication between pet owners, veterinarians and physicians.

## Supplementary Information

**Additional file 1: Table S1.** Number of eggs per gram (epg) for each helminth.

## Data Availability

The datasets supporting the conclusions of this article are included within the article. Due to commercial confidentiality of the research, data not included in the manuscript can only be made available to bona fide researchers subject to a non-disclosure agreement.

## Abbreviations

Epg: Eggs per gram
ESCCAP: European Scientific Council for Companion Animal Parasites
