# Supplementary material for: Prevalence of major digestive and respiratory helminths in dogs and cats in France: results of a multicenter study
Source: Parasit Vectors. 2022 Sep 6;15:314. doi: 10.1186/s13071-022-05368-7 (PMC9446561; doi:10.1186/s13071-022-05368-7)
Supplement: Supplementary file 1 — Additional file 1: Table S1. Number of eggs per gram (epg) for each helminth. [file 13071_2022_5368_MOESM1_ESM.docx]

**Additional file 1.**

**Table S1.** Number of eggs per gram (epg) for each helminth detected in dogs and/or cats.

| **Parasite species** | **Epg** | | | |
| --- | --- | --- | --- | --- |
|  | N | Mean | Median | 95% confidence interval |
| *Toxacara canis* | 35 | 646.3 | 84.0 | 6.55 - 4155.0 |
| *Toxacara cati* | 47 | 1327 | 500.0 | 14.15 -5897.0 |
| *Ancylostoma caninum* | 7 | 125.6 | 100.0 | 7.0 - 447.5 |
| *Uncinaria stenocephala* | 18 | 269.0 | 74.0 | 4.1 - 1598.1 |
| *Trichuris vulpis* | 11 | 150.2 | 15.0 | 7.0 - 725.0 |

*Dipylidium caninum* and Taeniidae has been identified only by the presence of white proglottis or by presence of eggs (without egg count).
